# Balancing Service and Leisure: Well-Being Outcomes of a Self-Funded Volunteering Experience in Zanzibar

**DOI:** 10.1192/j.eurpsy.2026.11831

**Published:** 2026-07-31

**Authors:** A. H. Almaghrebi

**Affiliations:** Psychiatry, King Saud Medical City, Riyadh, Saudi Arabia

## Abstract

**Introduction:**

Volunteering has been linked to improved mental health within the context of modern life, characterized by speed, rising demands, and constant pressures, yet most research focuses on students, older adults, or crisis contexts. Few studies examine self-funded humanitarian trips that blend meaningful service with leisure and cultural immersion among highly educated professionals.

**Objectives:**

This study explores whether balanced, self-funded volunteering enhances psychological well-being according to Ryff’s model, and whether the host country’s culture influences outcomes.

**Methods:**

This exploratory qualitative pilot study included 20 participants of diverse Arab nationalities and educational backgrounds who joined the *Travel with a Purpose* Foundation in Zanzibar to support school construction and medical clinics. One month after their return, participants were invited to answer 10 open-ended questions designed around their experience and guided by Ryff’s six dimensions. The questions explored psychological states, motivations, perceived changes, social media use, group dynamics, salient moments, and cultural influences. Responses were thematically analyzed and mapped to Ryff’s framework.

**Results:**

A total of 17 participants responded (mean age 32, 76.5% female); most held advanced degrees and prestigious positions. **Before the trip**, many described exhaustion, stress, or a sense of emptiness, while a few expressed motivation, anticipation, or coping with personal loss. Reflections pointed to difficulties with self-acceptance and environmental mastery. **During the trip**, participants reported feeling calmer, more present, and more emotionally regulated, often expressing gratitude, empathy, and courage. These changes mapped to mastery, positive relations, and personal growth. A total of 41% reduced their social media use, 24% stopped completely, and another 24% limited it to documenting their experience for family. A**fter the trip**, many described a renewed sense of balance and redefined priorities, linked to purpose in life, self-acceptance, and personal growth. More than half believed that self-funding increased commitment or improved logistics; for some, it also reflected autonomy. Impactful moments included interactions with children, teamwork, and witnessing local resilience, which deepened their appreciation for simplicity. Many explicitly attributed lasting effects to Zanzibar’s culture, epitomized by the phrase Hakuna Matata (“no worries”), which reflected a way of peaceful acceptance of circumstances.

**Conclusions:**

Findings suggest that balanced volunteer trips foster multiple Ryff dimensions of well-being, particularly for highly educated professionals working under high-pressure conditions. The host culture was also perceived as central in shaping outcomes. Due to the small sample size and short follow-up period, further research with larger, comparative samples is warranted.

**Disclosure of Interest:**

None Declared

